# Engineering biomimetic periosteum with β-TCP scaffolds to promote bone formation in calvarial defects of rats

**DOI:** 10.1186/s13287-017-0592-4

**Published:** 2017-06-05

**Authors:** Dan Zhang, Peng Gao, Qin Li, Jinda Li, Xiaojuan Li, Xiaoning Liu, Yunqing Kang, Liling Ren

**Affiliations:** 1 School of Stomatology, Lanzhou University, Lanzhou, Gansu 730000 China; 2 Department of Ocean and Mechanical Engineering, Florida Atlantic University, 777 Glades Road, Boca Raton, Florida 33431 USA; 3 Department of Biomedical Science, Florida Atlantic University, 777 Glades Road, Boca Raton, Florida 33431 USA

**Keywords:** Biomimetic periosteum, β-TCP, Cell sheet, Critical size defect, Tissue engineering

## Abstract

**Background:**

There is a critical need for the management of large bone defects. The purpose of this study was to engineer a biomimetic periosteum and to combine this with a macroporous β-tricalcium phosphate (β-TCP) scaffold for bone tissue regeneration.

**Methods:**

Rat bone marrow-derived mesenchymal stem cells (rBMSCs) were harvested and cultured in different culture media to form undifferentiated rBMSC sheets (undifferentiated medium (UM)) and osteogenic cell sheets (osteogenic medium (OM)). Simultaneously, rBMSCs were differentiated to induced endothelial-like cells (iECs), and the iECs were further cultured on a UM to form a vascularized cell sheet. At the same time, flow cytometry was used to detect the conversion rates of rBMSCs to iECs. The pre-vascularized cell sheet (iECs/UM) and the osteogenic cell sheet (OM) were stacked together to form a biomimetic periosteum with two distinct layers, which mimicked the fibrous layer and cambium layer of native periosteum. The biomimetic periostea were wrapped onto porous β-TCP scaffolds (BP/β-TCP) and implanted in the calvarial bone defects of rats. As controls, autologous periostea with β-TCP (AP/β-TCP) and β-TCP alone were implanted in the calvarial defects of rats, with a no implantation group as another control. At 2, 4, and 8 weeks post-surgery, implants were retrieved and X-ray, microcomputed tomography (micro-CT), histology, and immunohistochemistry staining analyses were performed.

**Results:**

Flow cytometry results showed that rBMSCs were partially differentiated into iECs with a 35.1% conversion rate in terms of CD31. There were still 20.97% rBMSCs expressing CD90. Scanning electron microscopy (SEM) results indicated that cells from the wrapped cell sheet on the β-TCP scaffold apparently migrated into the pores of the β-TCP scaffold. The histology and immunohistochemistry staining results from in vivo implantation indicated that the BP/β-TCP and AP/β-TCP groups promoted the formation of blood vessels and new bone tissues in the bone defects more than the other two control groups. In addition, micro-CT showed that more new bone tissue formed in the BP/β-TCP and AP/β-TCP groups than the other groups.

**Conclusions:**

Inducing rBMSCs to iECs could be a good strategy to obtain an endothelial cell source for prevascularization. Our findings indicate that the biomimetic periosteum with porous β-TCP scaffold has a similar ability to promote osteogenesis and angiogenesis in vivo compared to the autologous periosteum. This function could result from the double layers of biomimetic periosteum. The prevascularized cell sheet served a mimetic fibrous layer and the osteogenic cell sheet served a cambium layer of native periosteum. The biomimetic periosteum with a porous ceramic scaffold provides a new promising method for bone healing.

## Background

The healing of large bone defects is a significant clinical challenge in modern orthopedics. Although autologous bone grafts are a gold standard and allograft options are extensively used to treat bone defects in the clinical setting, they have their own drawbacks [1–3], including donor-side morbidity and increased operation time [4]. A synthetic bone substitute as an alternative solution holds great promise to treat large bone defects, and has been extensively studied over the past decades [5–7]. However, the clinical application of synthetic bone substitutes is still limited due to their insufficient vascularization capacity and limited bone-forming ability [8–10].

Periosteum is a thin film on the surface of the bone [11]. It is composed of two distinct layers. The external fibrous layer contains elastic fibers and microvessels, and the inner cambium layer contains periosteum-derived progenitor cells that play a crucial role in bone development and fracture healing [12, 13]. Studies have suggested that the periosteum is the main local source of skeletal stem/progenitor cells for bone healing [14]. In addition, periosteum also plays a key role in cell cytoskeletal reorganization [15]. Although autologous periosteum has shown promising potential in bone repair, the application of using autologous periosteum in bone regeneration is hindered due to its limited availability [16, 17]. There is therefore a critical need to develop an engineered biomimetic periosteum that can mimic the structure and function of native periosteum to enhance bone regeneration. Zhao et al. fabricated tissue-engineered periosteum by coupling either rabbit mesenchymal stem cells (MSCs) or differentiated MSCs with porcine small intestinal submucosa [18]. Shi et al. used MSCs and endothelial cell-laden porous collagen/nanobioactive glass partial composite scaffolds as the pseudo-periosteum [19]. Qi et al. seeded MSCs onto a human dermal fibroblast cell sheet for potential periosteum replacement [20]. However, these engineered periostea lack either blood vessels to support the tissue development or biodegradable porous scaffolds to form a three-dimensional (3D) biomimetic construct [21]. Therefore, the development of a functional periosteum which is similar to autologous periosteum in structure and function is much sort after. The biomimetic periosteum should have excellent osteogenic and angiogenic capability. Simultaneously, it should be able to integrate with scaffolds easily to match the geometrical contour of the defect.

Many cell therapies and tissue engineering approaches are seeking to mimic aspects of development to produce therapeutic cells or promote healing within specific microenvironmental contexts [22]. A novel MSC-based cell sheet engineering technique and its prevascularized cell sheet-based construct have been shown to have great potential in bone healing [23–25]. The composite cell sheets have been also used to create a 3D synthetic biomimetic-induced membrane (BIM) [14], which provided a better means for bone regeneration. In our previous study, we used a cell sheet engineering technique to prepare a biomimetic periosteum and wrapped it onto a beta-tricalcium phosphate (β-TCP) scaffold [21]. We further implanted the integrated periosteum/scaffold into the subcutaneous pockets of mice. We found that prevascularized periosteum promoted the anastomosis of the host vasculature and the scaffold supported the 3D biomimetic structure. However, whether the integrated biomimetic periosteum/scaffold can promote bone formation in an orthotopic bone site remains unknown.

Therefore, in this study we prepared two kinds of cell sheets to construct a biomimetic periosteum based on our developed cell sheet engineering technique. As the harvest of primary endothelial cells is still a challenge [26], we decided to use endothelial cell culture medium to differentiate rat bone marrow-derived MSCs (rBMSCs) into endothelial-like cells for the vascularization of the cell sheet. We prepared a prevascularized cell sheet and an osteogenic cell sheet, and then stacked the two cell sheets and wrapped them onto a small macroporous β-TCP disc to form a biomimetic periosteum/scaffold complex. Finally, we implanted the periosteum/scaffolds into calvarial defects of rats in vivo. We characterized the angiogenic and osteogenic ability of the combined periosteum/scaffolds.

## Methods

### rBMSC harvest and culture

The animal procedures were approved by the Institutional Medical Ethics Review Board of Lanzhou University School of Stomatology (LZUKQ20130305-2). Rat bone marrow was aspirated from the femoral medullary canal of Wistar rats (3–4 weeks old, 60–100 g) purchased from the Animal Experiment Center of Lanzhou University (Gansu province, China). The harvested bone marrow suspension was mixed with low-glucose Dulbecco's modified Eagle’s medium (L-DMEM; SH30021.01B, Hyclone, USA) supplement with 15% fetal bovine serum (FBS; Hyclone, USA) and 100 U/mL penicillin-streptomycin (Hyclone, USA). The culture medium was replaced every 2–3 days to remove nonadherent cells. After several medium changes, confluent cells were detached by 0.25% trypsin/EDTA and then further cultured in L-DMEM complete medium at 37°C in a 5% CO_2_ humidified incubator (Heraeus, Germany).

### Differentiation of rBMSCs into endothelial-like cells

rBMSCs were cultured at 3 × 10^4^ cells/cm^2^ in six-well plates with L-DMEM complete medium at 37°C in a 5% CO_2_ humidified incubator for 24 h, and then the L-DMEM medium was replaced by M199 medium (SH30253.01B, Hyclone, USA) with 10% FBS, 100 U/ml penicillin-streptomycin, 0.29 g/L Glutamine, 10 μg/L rat vascular endothelial growth factor (rVEGF; Peprotech, USA), and 2 μg/L rat basic fibroblast growth factor (rbFGF; Peprotech, USA) for 14 days. rBMSC was cultured in M199 complete medium without addition of rVEGF and rbFGF as a control.

### Flow cytometric analysis of the endothelial-like cells

After culture for 14 days, the differentiated rBMSCs were harvested using 0.25% trypsin/EDTA. A 100-μL cell suspension with a cell density of 1 × 10^5^/mL was transferred into an Eppendorf tube and then incubated with the following antibodies: a primary antibody CD31 (ab9498, Abcam, dilution 1:500) and PE anti-mouse CD90 (BD, USA) for 30 min at 4°C. A nondifferentiated rBMSC group served as a control. Then the stained samples were assessed by a flow cytometer (FACSVerse, BD, USA) and analyzed by FlowJo software. We designated the CD31-positive expression cells as induced endothelial cells (iECs). Platelet endothelial cell adhesion molecule (PECAM-1), also known as CD31, is a protein that is normally found on endothelial cells. CD90 can be used as a marker for a variety of stem cells.

### Production of a prevascularized cell sheet as the fibrous layer of the periosteum

**Fig. 1:**
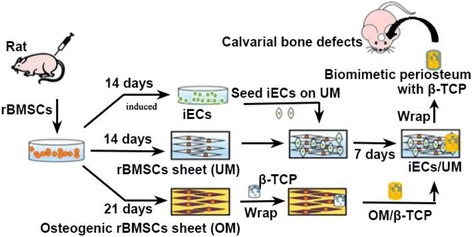
The schematic shows the whole procedure of the experiments. First, the rat bone marrow-derived mesenchymal stem cells (*rBMSCs*) were harvested and cultured in different culture media to form induced endothelial cells (*iECs*), pre-vascularized cell sheets (*iECs/UM*) and osteogenic cell sheets (*OM*). Then, these two types of cell sheets were cut into strips approximately 60 mm in length and 10 mm in width. The strip of the osteogenic cell sheet was wrapped on the beta-tricalcium phosphate (*β-TCP*) scaffold as the cambium layer of a native periosteum followed by the prevascularized cell sheet strip as the fibrous layer of a native periosteum. Thus, the BP/β-TCP constructs were formed. Finally, the BP/β-TCP constructs were implanted in the calvarial bone defect of a rat. *BP* biomimetic periosteum, *UM* undifferentiated medium

To engineer a prevascularized cell sheet in vitro, rBMSCs were first seeded on a 10-cm culture dish at a density of 1 × 10^5^/cm^2^ in L-DMEM complete medium. When rBMSCs reached confluence, the L-DMEM complete medium was changed to high-glucose DMEM (H-DMEM; SH30022.01B, Hyclone, USA), in which 50 mg/mL ascorbic acid was added to promote the production of extracellular matrix (ECM) [27]. After the rBMSCs were cultured for 14 days in H-DMEM, a dense, undifferentiated rBMSC sheet (UM) was formed. The endothelial differentiated rBMSCs (hybrid rBMSCs and iECs) were then seeded onto the UM sheet at a density of 5 × 10^4^/cm^2^ in a mixed medium which contained M199 and H-DMEM complete medium (1:1,v/v). Then the UM sheet with the hybrid rBMSCs and iECs was cultured for 14 days to produce a prevascularized cell sheet (iEC/UM). The experimental procedure is shown in Fig. 1.

### Immunofluorescent staining of the prevascularized cell sheet

After culture for 7 days, the prevascularized cell sheets (iEC/UM) were washed with phosphate-buffered saline (PBS), fixed in 4% paraformaldehyde for 15 min, and then blocked in a 5% goat serum-PBS buffer solution for 1 h at room temperature. A primary antibody rabbit anti-mouse CD31 (ab124432, Abcam, dilution 1:500) in 1% bovine serum albumin (BSA)-PBS was added to the samples and incubated overnight at 4°C. After washing with PBS, a secondary antibody goat anti-rabbit (Alexa Fluor 594, 2 μg/mL, Invitrogen) in 1% BSA-PBS buffer was added and incubated in the dark for 1 h at room temperature. Finally, the fluorescent staining images were captured by confocal microscopy (Research inverted system microscope, IX71, Olympus).

### Production of an osteogenic cell sheet as the cambium layer of a periosteum

Besides the prevascularized cell sheet, the osteogenic cell sheet was fabricated at the same time. rBMSCs were cultured in an osteogenic medium which contained 10% FBS, 10 mM β-glycerophosphate, 10 nM dexamethasone, and 50 mg/L ascorbic acid for 21 days to form an osteogenic differentiated cell sheet (OM). To characterize its osteogenic properties, alkaline phosphatase (ALP) and alizarin red staining were performed.

### Preparation of BP/β-TCP constructs

The porous β-TCP scaffolds were prepared according to our previously published method [21]. The diameter of the scaffold was approximately 8 mm and the thickness was 1.5 mm. This dimension was appropriate for the specific application in the calvarial implantation. The pore size was around 350–500 μm and the average porosity was nearly 80% according to our published method [21]. After ultrasonic cleaning (KQ-250DB, Kunshan, China), β-TCP scaffolds were sterilized and stored for further use (Vertical Heating Pressure Steam Sterilizer, Shanghai, China). Figure 1 showed the procedure of producing the biomimetic periosteum (BP)/β-TCP constructs. First, we cultured cells to form prevascularized cell sheets (iEC/UM) and osteogenic cell sheets (OM). The two types of cell sheets were then cut into strips approximately 60 mm in length and 10 mm in width. The strip of OM was then firstly wrapped on the β-TCP scaffold as the cambium layer of the native periosteum followed by the iEC/UM strip as the fibrous layer. Thus, the BP/β-TCP constructs were formed.

At the same time, we prepared autologous periostea (APs) from rats when we began the animal experiments. After the rat calvarium was exposed, normal saline was injected between the calvarium and periosteum in order to separate them easily. Afterwards, forceps were gently clamped to one side of a periosteum, and a scissor was used to cut the other side of the periosteum. The periosteum was gently peeled off the calvarium. After washing in a saline buffer, the autologous periosteum was wrapped on a β-TCP scaffold to form a combined autologous periosteum/scaffold (AP/β-TCP). The AP/β-TCP was implanted into a calvarial bone defect in vivo under sterile conditions.

### In vivo implantation

The in vivo animal implantation method was approved by the Institutional Medical Ethics Review Board of Lanzhou University School of Stomatology (LZUKQ20130305-2). The adult healthy female Wistar rats (180–220 g) were purchased from the Animal Experiment Center of Lanzhou University, Gansu province, China. Rats were anesthetized by intraperitoneal injection of 10% chloral hydrate (4 mL/kg body weight) and fixed on the board. After an aseptic preparation was applied to the skin, a 2-cm semilunar incision was made through the skin and the muscle down to the cranial vertex. After exposing the calvarium, an 8-mm diameter bone defect was created using a 5 mm-diameter round bur under continuous saline buffer irrigation. Sixty rats were randomly allocated into the following groups at three time points (2, 4, and 8 weeks): 1) BP/β-TCP (*n* = 5); 2) AP/β-TCP (*n* = 5); 3) plain β-TCP (*n* = 5); and 4) no implantation group (*n* = 5). Wounds were closed and sutured, and animals were housed for the designated time according to the experimental period.

### X-ray and micro-CT scanning

At 4 or 8 weeks post-surgery, the animals were anesthetized and calvarial samples were harvested and fixed in 4% paraformaldehyde for 3 days. Mineral formation within the defect area and the state of the implanted β-TCP scaffolds were evaluated by a dental digital X-ray machine (60–70 kV, 7 mA, Minary, SOREDEX, Finland) and microcomputed tomography (micro-CT; Siemens Inveon, Germany) at a resolution of 40 μm. Finally, each sample at 8 weeks was reconstructed and analyzed by Mimics10.01 software.

### Histology and immunohistochemistry staining

At 2, 4, and 8 weeks, the implanted samples were carefully retrieved, washed in PBS, fixed in 4% paraformaldehyde for 3 days, and decalcified in 50 mM EDTA for 2–4 weeks at room temperature. We chose 8-week skull samples to measure the newly formed bone volume because there would be a greater quantity of new calcified osteoid matrix than the degradation of the scaffold according to our previous study [21]. The samples were gradually dehydrated in a serial gradient alcohol from 70% to 100%, embedded in paraffin, and then cut into 5-μm sections. Conventional hematoxylin and eosin (H&E) and Van Gieson’s staining were carried out on the sections. Five sections of each group were used for histometric analysis of newly formed bone tissue based on the images of Van Gieson’s staining. We quantified the pixels of the dense red color of the stained images. The increased volume ratio of newly formed bone to the entire tissue in the implants was measured by Origin 7.0.

The immunohistochemical staining of anti-rat CD31 was performed on the sectioned slices. The samples were deparaffinized and digested by an antigen retrieval solution, and then the sections were blocked by blocking serum (5%) for 30 min at room temperature. The sections were incubated with primary antibody rabbit anti-mouse CD31 (ab124432 Abcam, dilution 1:500), and then the secondary antibody goat anti-rabbit (Alexa Fluor 594, 2 μg/mL, Invitrogen) was added to stain the samples. At the same time, a DAB substrate kit (Vector Laboratories) was used followed by hematoxylin counterstaining and permanent mounting. The microvessels formed in the defect area were quantified by CD31-positive expression quantities by using Image J software.

### Statistical analysis

Numerical data are expressed as mean value ± SD and the statistical significance was assessed by analysis of variance (ANOVA) and Tukey post-hoc tests using SPSS 17.0 software (SPSS), where a significant difference was considered if the *p* value was less than 0.05.

## Results

### Cell morphology and fabrication of cell sheets

**Fig. 2:**
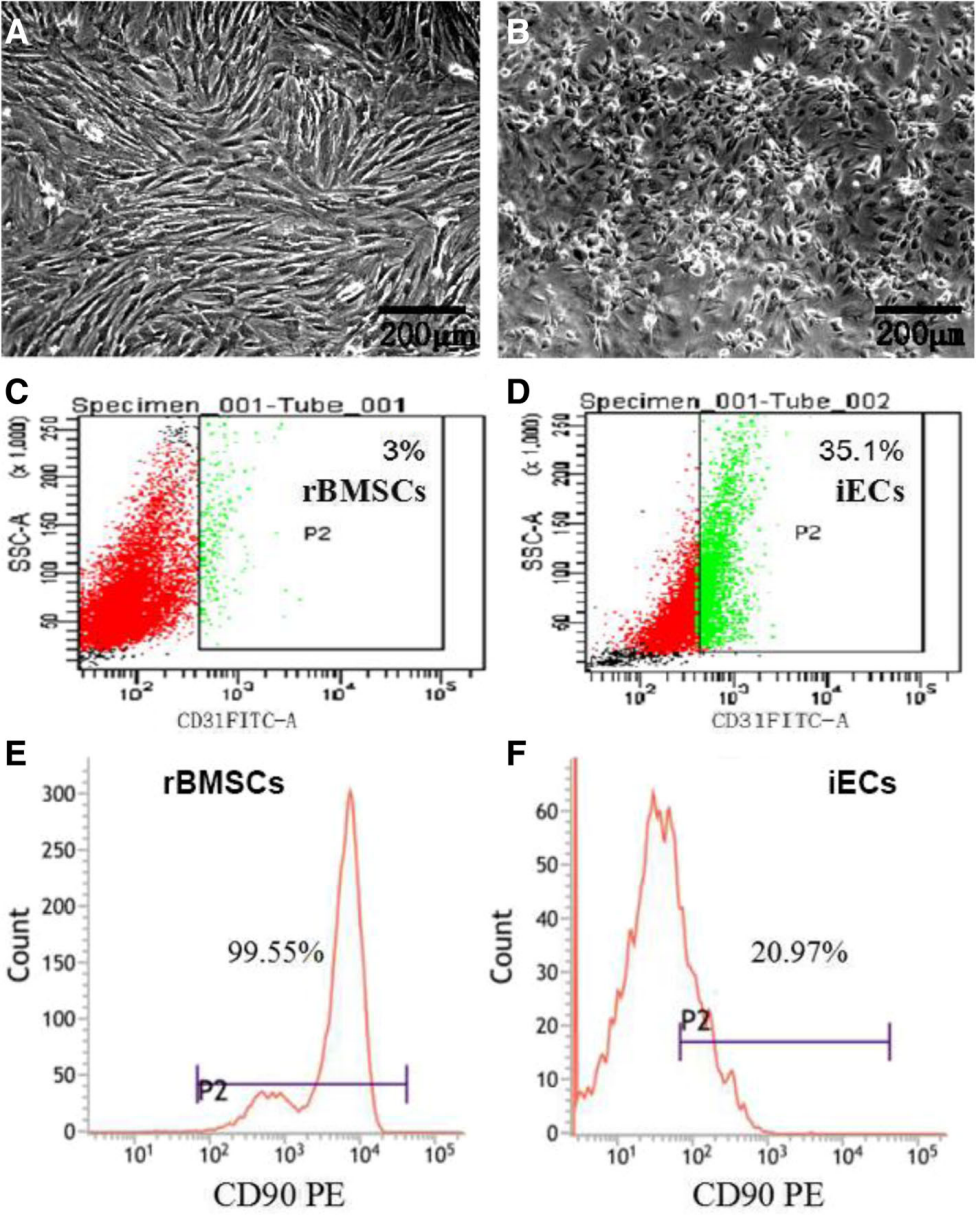
Cell morphologies of rat bone marrow-derived mesenchymal stem cells (*rBMSCs*) (**a**) and induced endothelial cells (*iECs*) (**b**). Nondifferentiated rBMSCs show a typical spindle shape, while iECs are cobblestone-like and oval. Flow cytometric analysis of CD31 of nondifferentiated rBMSCs (**c**) and iECs (**d**). Flow cytometric analysis of CD90 of nondifferentiated rBMSCs (**e**) and iECs (**f**). The results indicate that rBMSCs have the ability to differentiate into endothelial cells under experimental conditions. *Scale bars* = 200 μm

Figure 2 showed cell morphologies of nondifferentiated rBMSCs (Fig. 2a) and iECs (Fig. 2b). It can be seen that rBMSCs showed a spindle shape and were spreading, while the iECs were oval and displayed a typical cobblestone-like morphology. Flow cytometry results showed that nondifferentiated rBMSCs expressed only 3% CD31 (Fig. 2c) but, after they were incubated in the endothelial culture medium, 35.1% rBMSCs had expressed CD31^+^ (Fig. 2d). We further characterized the stem cell marker CD90. Results indicated that 99.55% nondifferentiated rBMSCs expressed CD90 (Fig. 2e), but only 20.97% rBMSCs expressed CD90 after they were cultured in the endothelial culture medium (Fig. 2f).

**Fig. 3:**
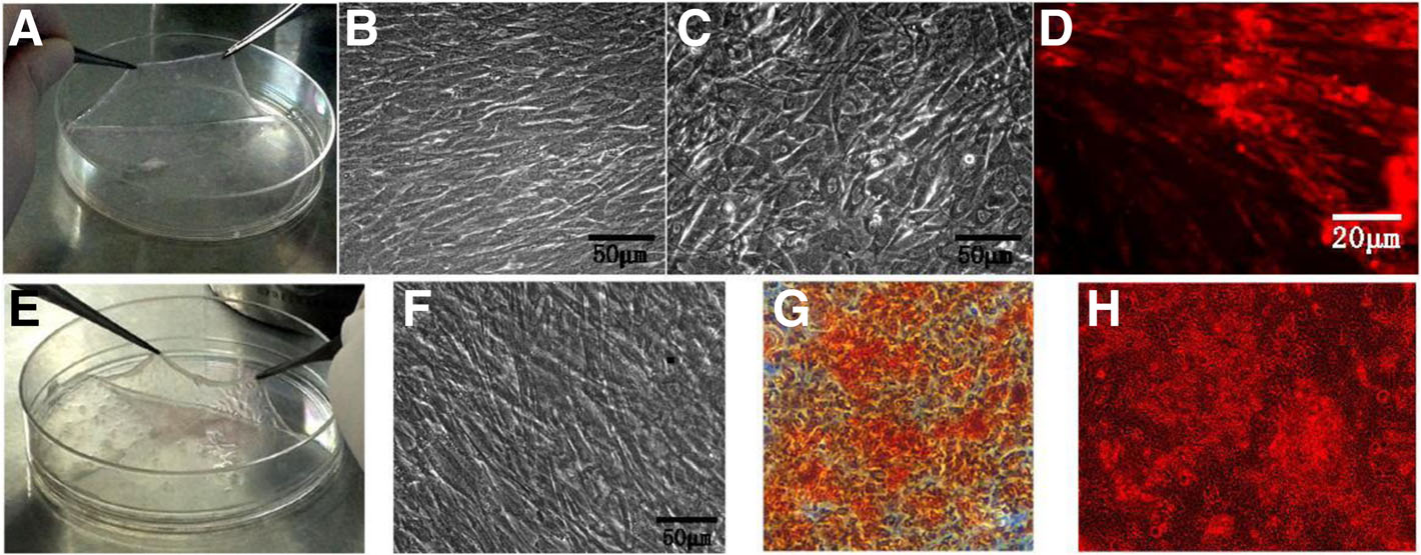
The view and microscopic morphology of UM (**a**, **b**) and OM (**e**, **f**), which show the cell sheets formed multiple layers and can be lifted up by point forceps; when iECs were seeded in the UM at day 7 (**c**), the UM morphology is significantly changed. Immunofluorescent staining of CD31 showed the in vitro angiogenesis of the prevascularized cell sheet (iECs/UM) (**d**). ALP at day 14 (**g**) and alizarin red staining at day 21 (**h**) on the OM sheet show obvious osteogenic differentiation characteristics of the OM sheet. *Scale bars* = 50 μm (**b**,**c**,**f**) and 20 μm (**d**)

Figure 3a shows the gross view of the undifferentiated rBMSC sheets (UM). The cell sheet is shown as being lifted by two forceps for the next step (Fig. 3a). Microscopic morphology showed that rBMSCs grew and formed a dense matrix (Fig. 3b). After the differentiated rBMSCs (iECs) were seeded and cultured on the UM sheet, the microscopic morphology was significantly changed (Fig. 3c). Immunofluorescent staining of CD31 was performed to investigate the in vitro angiogenesis of the prevascularized cell sheet (data not shown). Figure 3d shows the formation of progressive and rich branched networks in the UM cell sheet at day 7. After the rBMSCs were cultured in osteogenic medium for 21 days, the rBMSCs were differentiated and formed an osteogenic differentiated rBMSC (OM) cell sheet. The OM cell sheet is shown as being lifted up using two forceps (Fig. 3e). The cell sheets showed multilayers with a slight light reflection. Figure 3f shows that the microscopic morphology of the osteogenic cell sheet was remarkably different from that of the UM cell sheet (in Fig. 3b). The undifferentiated rBMSCs in the UM sheet showed that the cells were typically spindle-shaped, while the rBMSCs in the OM sheet were cuboidal and short. Both ALP at day 14 (Fig. 3g) and alizarin red staining at day 21 (Fig. 3h) show that the OM sheet had obvious osteogenic differentiation characteristics.

### Characteristics of the BP/β-TCP complex

**Fig. 4:**
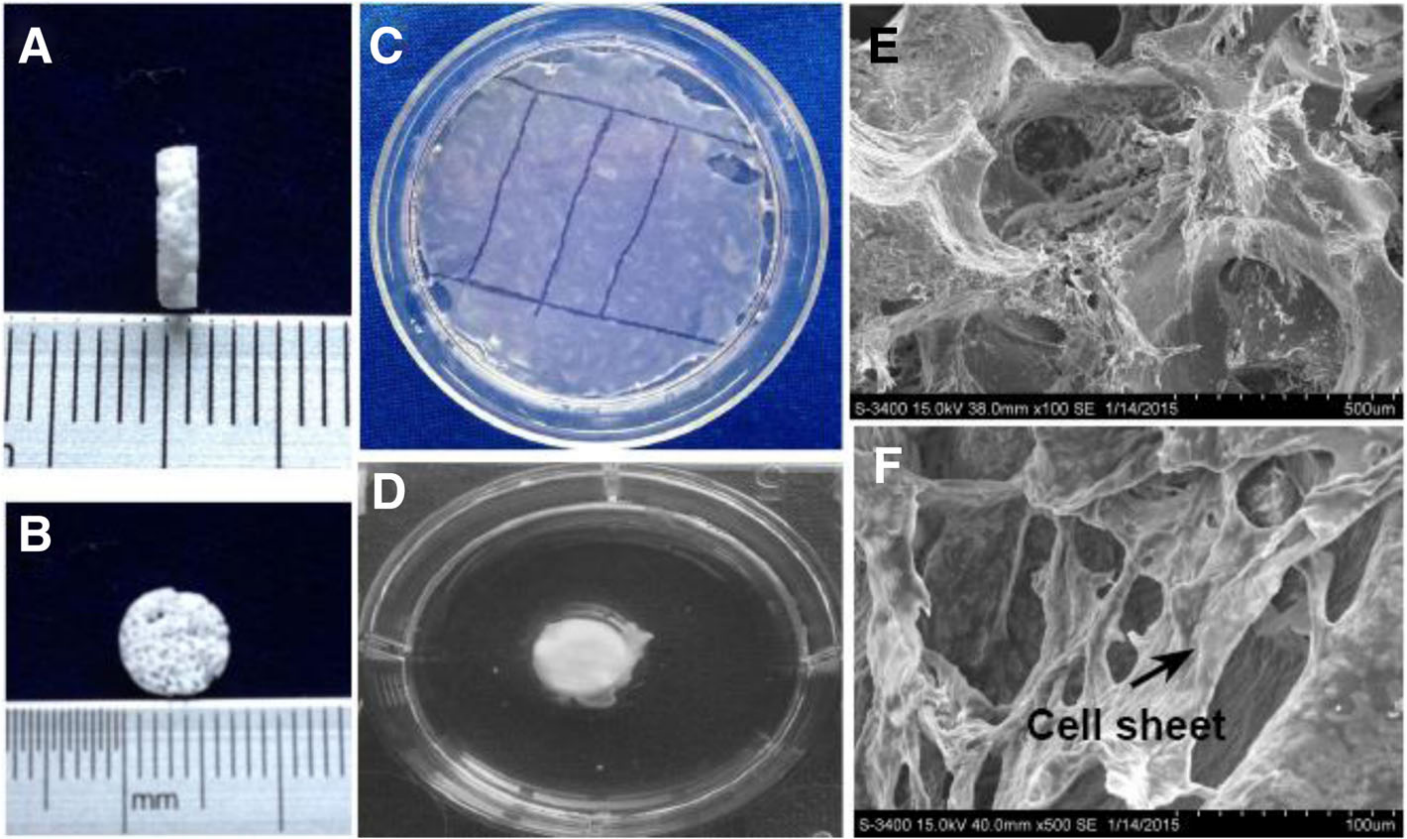
The β-TCP scaffold was approximately 8 mm in diameter and 1.5 mm in thickness (**a**,**b**). The OM and iEC/UM sheets were cut into strips approximately 60 mm in length and 10 mm in width, then wrapped onto the β-TCP scaffold, thus generating the BP/β-TCP (**c**,**d**). SEM images of BP/β-TCP indicated that the cells from the cell sheet apparently migrated into the porous β-TCP scaffold and that rich ECM surrounded the scaffold (**e**,**f**). *Scale bars* = 500 μm (**e**) and 100 μm (**f**)

In this study, we used our porous β-TCP scaffolds to construct the biomimetic periosteum. The β-TCP scaffold was 8 mm in diameter and 1.5 mm in height (Fig. 4a and b), and had interconnected macropores. The cell sheets were cut into strips approximately 60 mm in length and 10 mm in width (Fig. 4c). The strips of the cell sheet were then wrapped on the scaffold (Fig. 4d). After the wrapped cell sheets with β-TCP were cultured for 3 days, scanning electron microscope observation was performed and the results indicated that the cells from the cell sheet apparently migrated into the porous β-TCP scaffold, which was also surrounded by the rich ECM (Fig. 4e and f).

### In vivo implantation

**Fig. 5:**
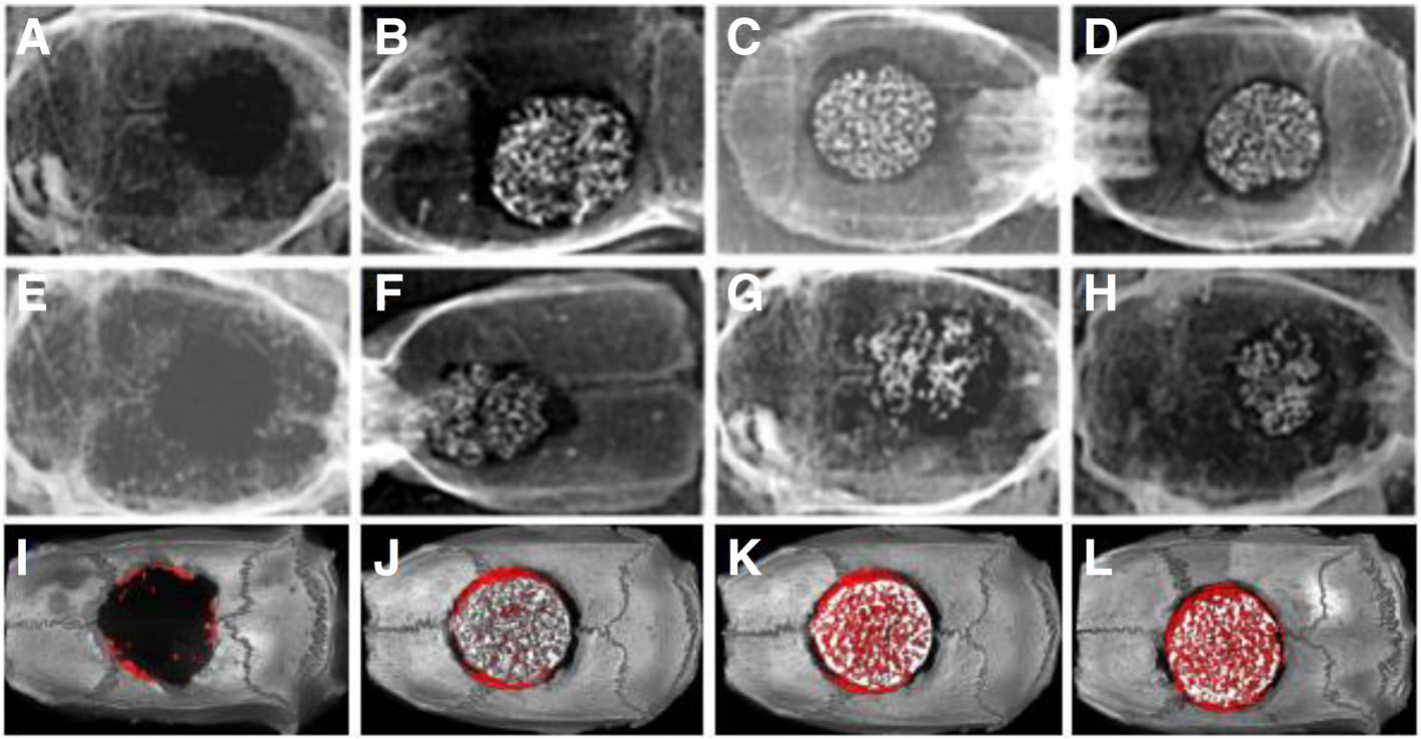
X-ray images of the implanted specimens after 4 weeks: no implantation group (**a**), plain β-TCP (**b**), AP/β-TCP (**c**), and BP/β-TCP (**d**). X-ray images of the implanted specimens after 8 weeks: no implantation group (**e**), plain β-TCP (**f**), AP/β-TCP (**g**), and BP/β-TCP (**h**). With time, bone formation was obviously observed at 4–8 weeks with evidence of the gradual decrease in the defect gap and the increase in the volume of new bone. Micro-CT images of the implanted specimens after 8 weeks: no implantation group (**i**), plain β-TCP (**j**), AP/β-TCP (**k**), and BP/β-TCP (**l**). The *red color* represents the newly formed bone tissue

The BP/β-TCP were implanted into rat calvarial bone defects with a diameter of 8 mm. Figure 5 shows the X-ray macroscopic view of specimens after implantation for 4 and 8 weeks (Fig. 5a–h). From the results of the macroscopic view of each specimen at 4 weeks, we can see that the β-TCP scaffolds did not obviously absorb, but the β-TCP and the surrounding tissues had a tight connection compared to the no-implantation group (Fig. 5c and d). At 8 weeks, the β-TCP scaffolds in the BP/β-TCP and AP/β-TCP groups had degraded partially. The scaffold and surrounding tissues showed a strong connection (Fig. 5g and h). With time, the sizes of the defects became smaller and the β-TCP scaffolds gradually degraded. Micro-CT results at 8 weeks further show that the volume of newly-formed bone (the red color) in the BP/β-TCP and AP/β-TCP groups (Fig. 5k and l) had obviously increased more than the other two groups (Fig. 5i and j).

### Evaluation of the vascular network and osteogenesis in vivo

**Fig. 6:**
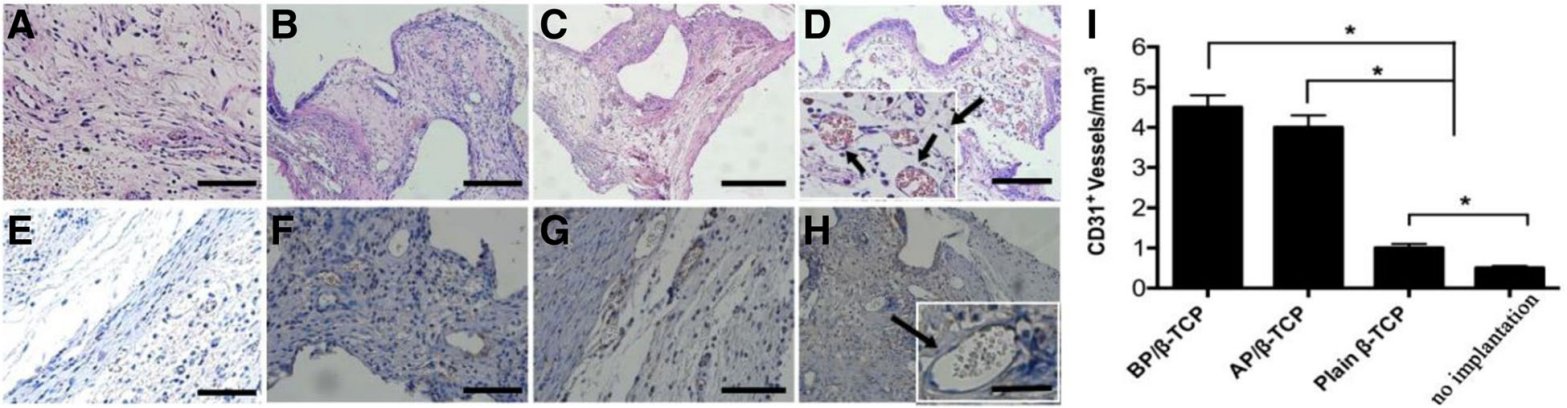
H&E staining on the sectioned slices at 2 weeks showing that the plain β-TCP (**a**) and no-implantation group (**b**) had hardly any lumen formation; however, numerous lumens containing murine erythrocytes in the AP/β-TCP group (**c**) and BP/β-TCP group (**d**) were observed. Similar results were seen in the immunohistochemical staining of CD31 on the four groups: plain β-TCP (**e**), no-implantation group (**f**), AP/β-TCP (**g**), and BP/β-TCP group (**h**). Two magnified images in the BP/β-TCP group clearly show the lumens containing murine erythrocytes (*scale bar* = 100 μm). Quantitative results (**i**) show a significant difference in the vascular volume among the periosteum groups and nonperiosteum group (*p* < 0.05) by counting CD31-positive expressing lumens, while there was no significant difference between the BP/β-TCP and AP/β-TCP groups(*p* < 0.05). *β-TCP* beta-tricalcium phosphate, *AP* autologous periosteum, *BP* biomimetic periosteum

**Fig. 7:**
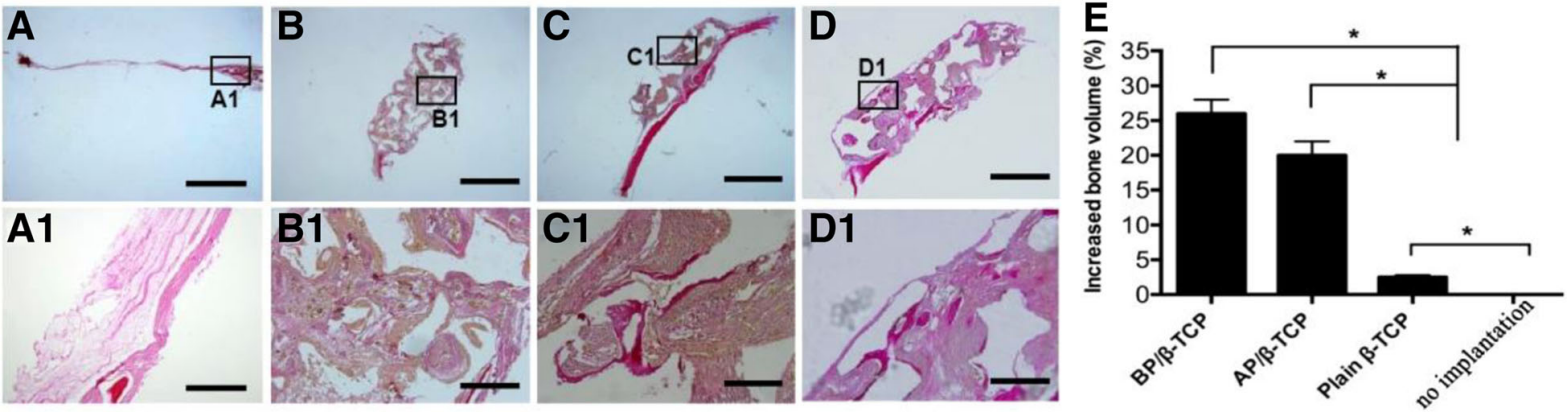
Van Gieson's staining on the sections of the four groups at 8 weeks showed that there was little calcified osteoid matrix (*dense red* color) formed in the no-implantation group (**a**,**a1**) and plain β-TCP (**b**,**b1**) scaffolds. However, in the BP/β-TCP (**d**,**d1**) and AP/β-TCP (**c**,**c1**) groups, more newly calcified osteoid matrix and bone formation were observed. (A ~ D: Scale bars = 1 mm (**a**–**d**) and 100 μm (**a1**–**d1**). **e** Quantitative results of the increased bone volume show that the volumes of calcified matrix in the BP/β-TCP complex group or the AP/β-TCP group were significant higher than those in the plain β-TCP scaffolds and no-implantation group (*p* < 0.05). *β-TCP* beta-tricalcium phosphate, *AP* autologous periosteum, *BP* biomimetic periosteum

CD31 immunohistochemical staining and H&E staining at 2 weeks was performed to investigate the formation of blood vessels, defined as lumens with murine erythrocytes in the grafts. The results show that there were few lumens formed in the plain β-TCP group and the no-implantation group (Fig. 6a, b, e, and f). However, numerous lumens containing murine erythrocytes were observed in the AP/β-TCP group (Fig. 6c and g) and the BP/β-TCP group (Fig. 6d and h). Additionally, two magnified images from the BP/β-TCP complex group clearly showed the lumens containing murine erythrocytes (Fig. 6d and h), which indicated that the prevascularization in vitro promoted the invasion of the host vascular system into the implants. Quantitative results (Fig. 6i) showed a significant difference in the vascular volume among the periosteum groups and nonperiosteum group (*p* < 0.05) by counting CD31-positive expressing lumens. There was no significant difference between the BP/β-TCP and AP/β-TCP groups (*p* < 0.05). Van Gieson’s staining was performed to study the mineral volume at the period of 8 weeks. Figure 7 indicates the osteoblast activity of each group. Results showed that there was little calcified osteoid matrix (dense red color) formed in the no-implantation group and plain β-TCP scaffold group (Fig. 7a and b). However, in the BP/β-TCP and AP/β-TCP groups, more newly calcified osteoid matrix and bone formation were observed. Figure 7e shows the quantification of the calcified osteoid matrix based on the red stained area. Results show that the volumes of formed calcified matrix in the BP/β-TCP and AP/β-TCP groups were similar. There was no significant difference between these two groups. The volumes of calcified matrix in the BP/β-TCP complex group or the AP/β-TCP group were significant higher than those in the plain β-TCP scaffold group and no-implantation group (*p* < 0.05). All in all, the results indicated that the BP/β-TCP group can promote the formation of calcified matrix compared with the plain group and no-implantation group.

## Discussion

In this study we used a cell sheet engineering technique to develop vascularized and osteogenic cell sheets. We then combined them with a macroporous β-TCP scaffold to construct a biomimetic structure of the periosteum for potential application in the regeneration of large bone defects. We found that the biomimetic periosteum had similar ability to promote osteogenesis in vivo compared to the autologous periosteum.

To mimic the anatomical structure of the native periosteum, we wrapped the OM cell sheet onto the porous β-TCP scaffold, which mimicked the inner cambium layer of native periosteum, followed by wrapping the vascularized UM cell sheet which mimicked the outer fibrous layer. The β-TCP scaffold was a mechanical supporter of these two layers, with which a biomimetic periosteum-covered bone-like graft was constructed. We found that the BP/β-TCP had great potential in promoting vascularization and osteogenesis in vivo. It demonstrated similar function to the autologous periosteum. These results also further confirm the regenerative function of the biomimetic periosteum in our previous study [28]. Our previous study reported that the combination of a prevascularized biomimetic periosteum cell sheet and a β-TCP scaffold not only promoted the formation and stability of the blood vessel network, but also provided an osteoblast source to enhance bone formation in an ectopic site [21]. In this study, the similarly structured biomimetic periosteum combined with the scaffold further confirmed its promising regenerative ability in an orthotopic site.

Prevascularization is a promising strategy to promote the in vivo vascularization of a synthetic scaffold. The strategy has been widely demonstrated to promote the formation of functional vascular networks and is rapidly anastomosed with the host vascular system [15, 23]. A critical consideration for in vitro prevascularization is the use of endothelial cells. It is challenging to obtain an abundant source of efficient autologous endothelial cells [29–31]. Studies have shown that MSCs have the multipotent ability to differentiate into osteoblast and endothelial cells [23, 27, 32]. Therefore, in this study we used rVEGF and rbFGF to induce rBMSCs to iECs to solve the problem of the cell source of endothelial cells. Divya et al. successfully induced porcine mesenchymal stem cells to endothelial cells, which provided new options for re-endothelialization [33]. Wang et al. suggested that VEGF induced human and rat bone marrow-derived MSCs to endothelial cells by Rho/ROCK signaling-mediated nuclear translocation of MRTF-A [34]. Liu et al. demonstrated that the co-culture rabbit MSC-derived endothelial cells improved the osteogenesis of MSCs and promoted new bone formation [35]. Our in vitro results indicated that the morphology of induced rBMSCs changed from the spindle shape of nondifferentiated rBMSCs to cobblestone endothelial-like morphology after culture for 14 days. Flow cytometric analysis implied that the rBMSCs had the ability to differentiate into endothelial cells under the experimental conditions. At the same time, results showed that the percentage of rBMSCs with the mesenchymal phenotype decreased from around 99% to 21%. Approximate 78% of rMBSCs lost the mesenchymal phenotype as seen by the negative expression of CD90. However, only 35.1% of rBMSCs had differentiated to CD31^+^ cells. This result implied that around 43% of cells did not demonstrate either the mesenchymal phenotype (CD90^+^) or the endothelial phenotype (CD31^+^). We did not further verify to which cell type the fraction of the 43% cells belonged. They may be in a transition state from the mesenchymal to endothelial phenotype. Due to the hybrid state of the endothelial-differentiated rBMSC/iEC populations, we did not sort the pure iECs from the hybrid populations for the prevascularized cell sheet. Therefore, it is worth noting that the seeded iECs on the UM sheet (Fig. 1) were not pure endothelial cells but hybrid endothelial-differentiated rBMSC/iEC populations. Even so, the in vivo results still showed that the amount of blood vessels containing murine erythrocytes formed in the BP/β-TCP complex group was similar to that in the AP/β-TCP group. This result further confirmed that inducing BMSCs into vascular endothelial cells may bring a new endothelial cell source for tissue engineering.

Scaffolds for load-bearing application in bone regeneration should be mechanically stable to provide mechanical support, as well as being bioactive, facilitating or initiating proliferation and osteogenic differentiation of cells, ECM production, and eventually bone deposition [36]. In addition, we used a mechanically sound biodegradable porous β-TCP scaffold to support the transplantation of the biomimetic periosteum. Results demonstrated a significant increase in osteogenesis and angiogenesis in the scaffolds over time. Most of the 3D scaffolds for bone healing have limited tissue ingrowth due to restrained nutrient supply imposed by intrinsic geometrical and structural characteristics [37]. β-TCP has been used to fabricate scaffolds owing to its good osteoconductivity and degradation [38, 39]. The β-TCP scaffold with interconnected pores was sequentially wrapped by the osteogenic cell sheet and prevascularized cell sheet layer by layer to fabricate engineered periosteum, which enlarged the area of cell adhesion, proliferation, and tissue ingrowth, as well as the subsequent ECM formation. The biomimetic periosteum composed of the prevascularized cell sheet and osteogenic rBMSC sheet created the concomitant regeneration of vasculature and new bone tissue. This approach provides a new strategy for bone tissue regeneration. In conclusion, biomimetic periosteum combined with β-TCP holds promising potential to be used in bone tissue engineering as bone substitutes.

## Conclusion

The successful induction of rBMSCs into endothelial-like cells provided the endothelial cell source for angiogenesis. The production of a prevascularized cell sheet as the fibrous layer and osteogenic cell sheet as the cambium layer of the periosteum not only provided a source of skeletal stem/progenitor cells for bone healing, but also contained a rich ECM for efficiently promoting the formation of a functional vessel system and new bone regeneration. The mechanical supports of the scaffold also successfully realized the function of the biomimetic periosteum. The biomimetic periosteum mimicking the structure of a native periosteum showed great potential in promoting vascularization and osteogenesis in vivo, when supported by a porous β-TCP scaffold. This strategy provides promising potential to regenerate bone defects.

## Abbreviations

3D: Three-dimensional
β-TCP: Beta-tricalcium phosphate
ALP: Alkaline phosphatase
AP: Autologous periosteum
BP: Biomimetic periosteum
BSA: Bovine serum albumin
ECM: Extracellular matrix
FBS: Fetal bovine serum
H-DMEM: High-glucose Dulbecco’s modified Eagle’s medium
H&E: Hematoxylin and eosin
iEC: Induced endothelial cell
L-DMEM: Low-glucose Dulbecco’s modified Eagle’s medium
micro-CT: Microcomputed tomography
MSC: Mesenchymal stem cell
OM: Osteogenic medium
PBS: Phosphate-buffered saline
rbFGF: Rat basic fibroblast growth fator
rBMSC: Rat bone marrow-derived mesenchymal stem cell
rVEGF: Rat vascular endothelial growth factor
UM: Undifferentiated medium
